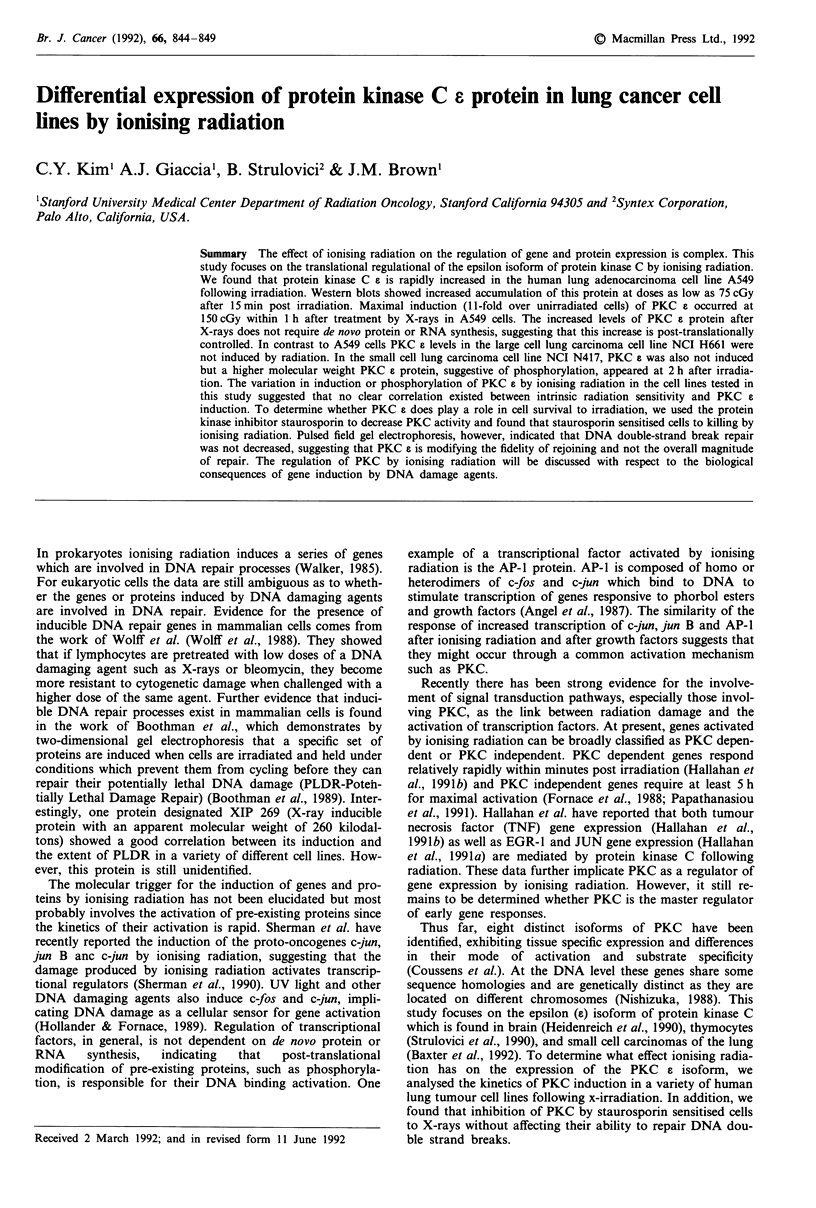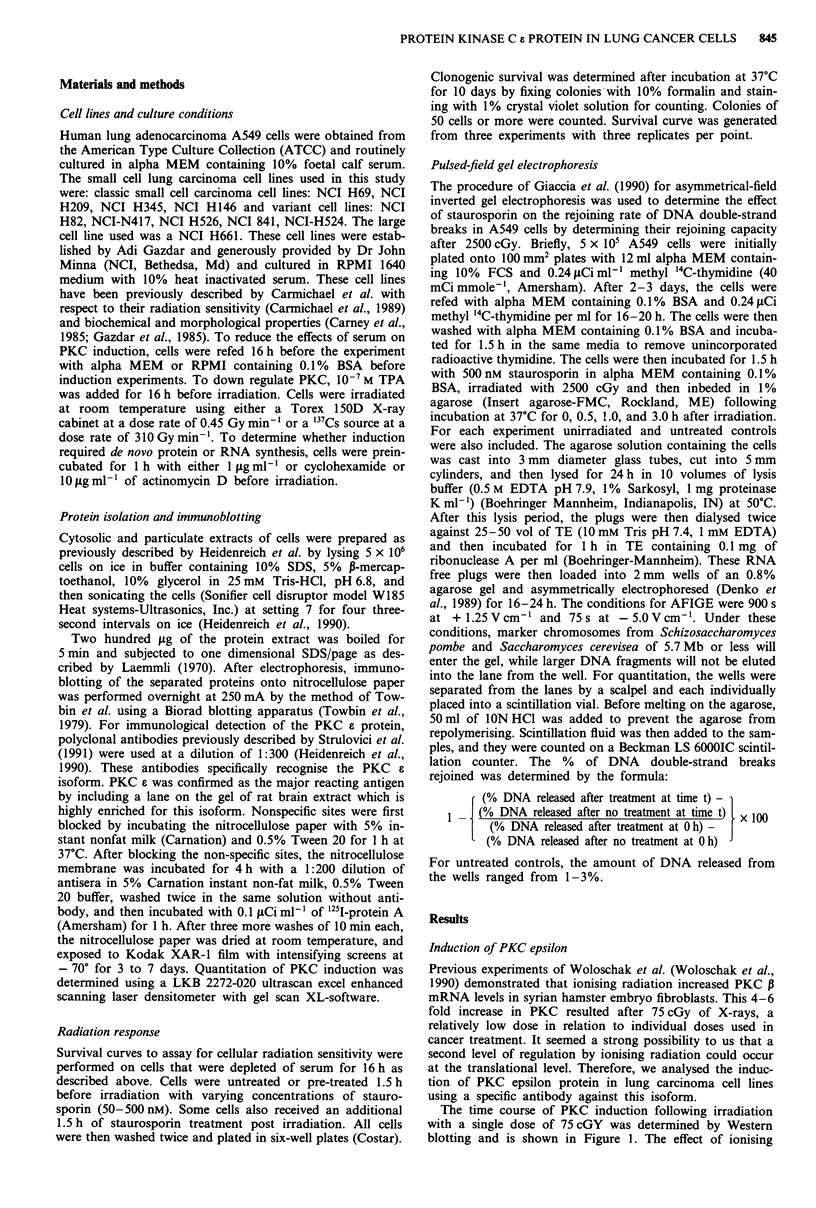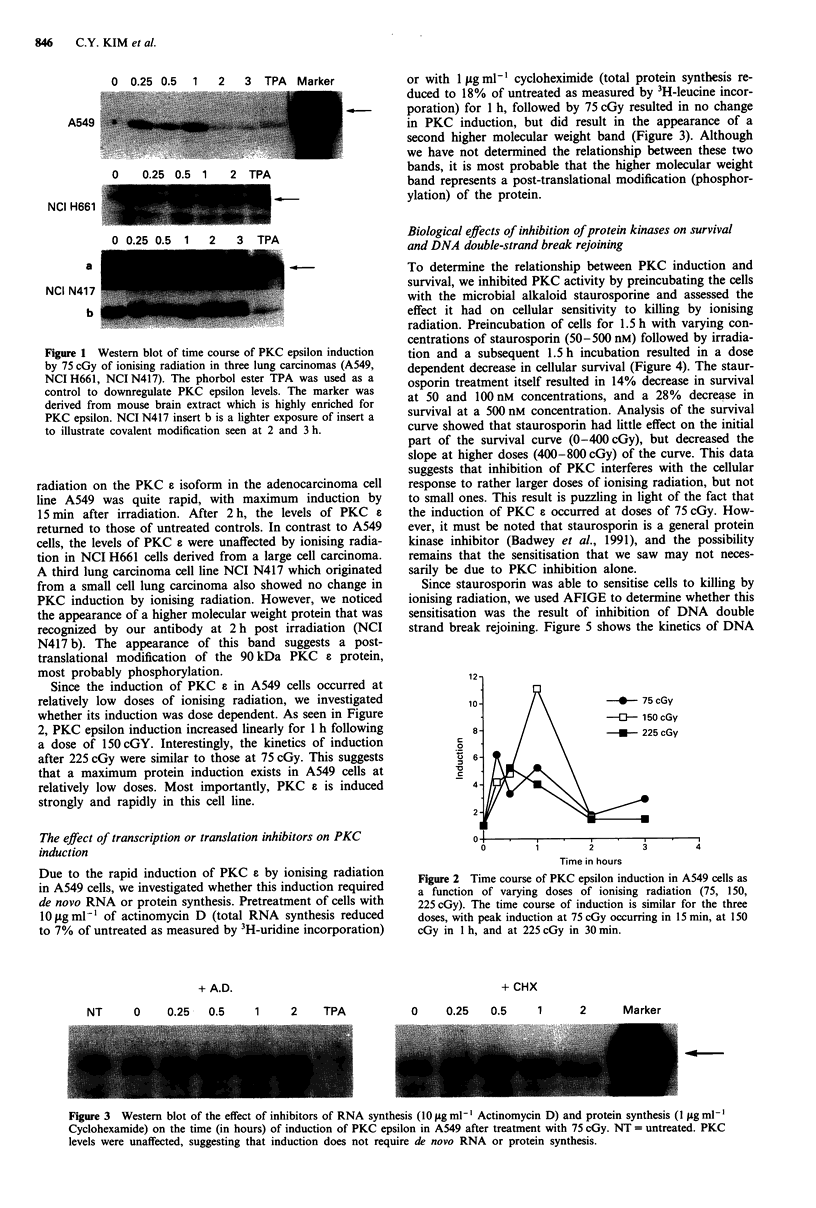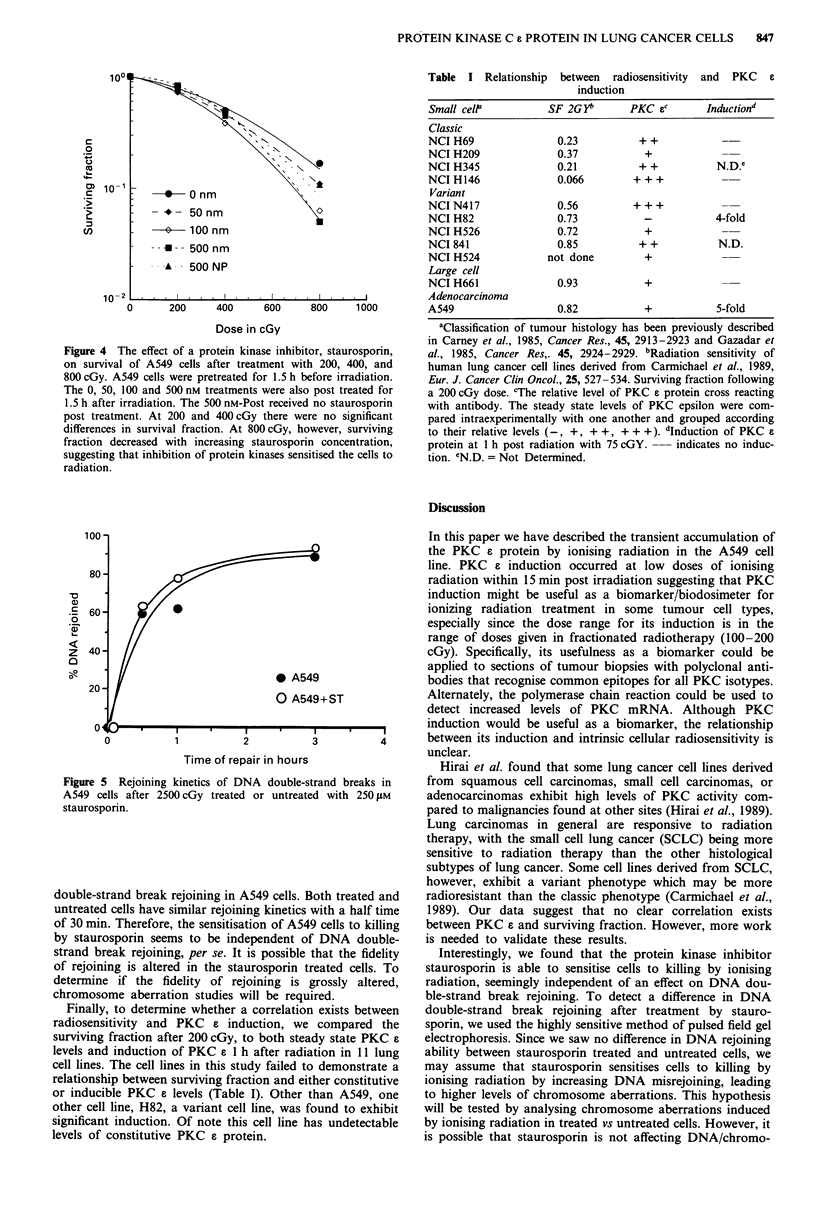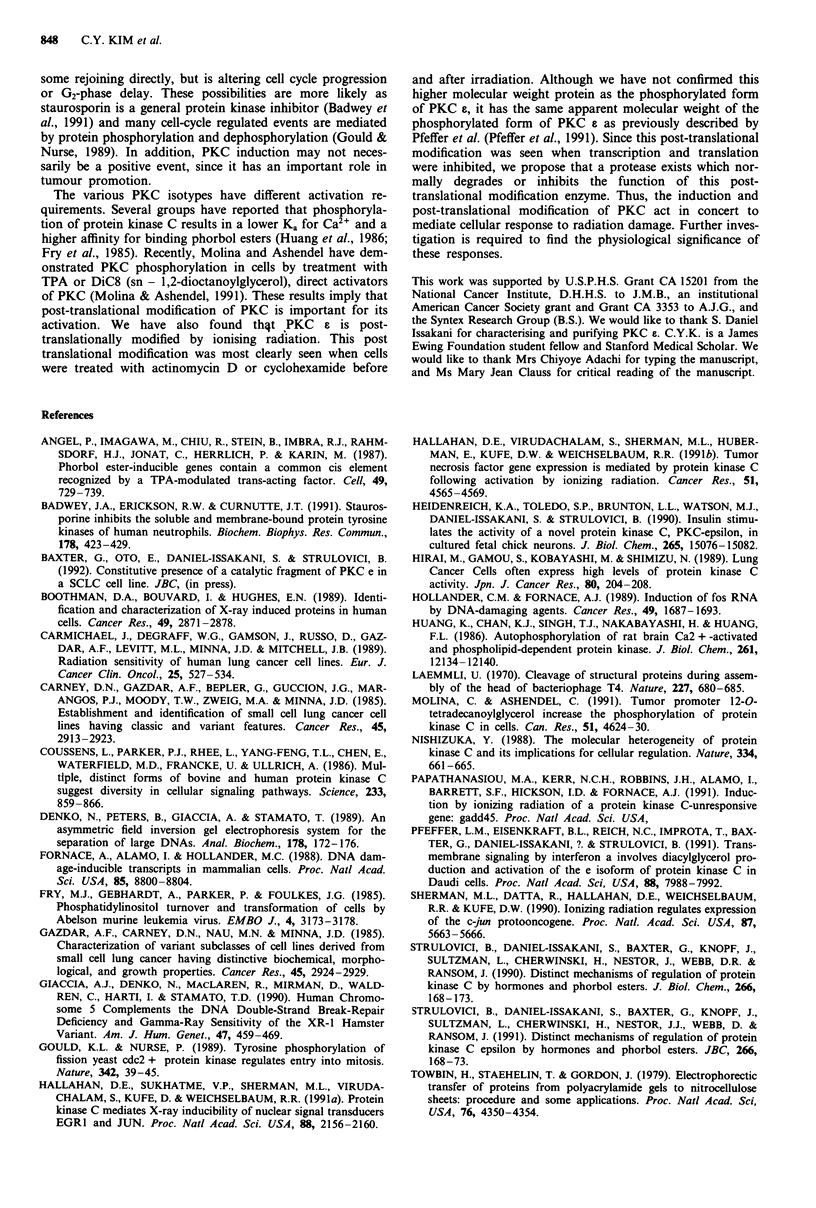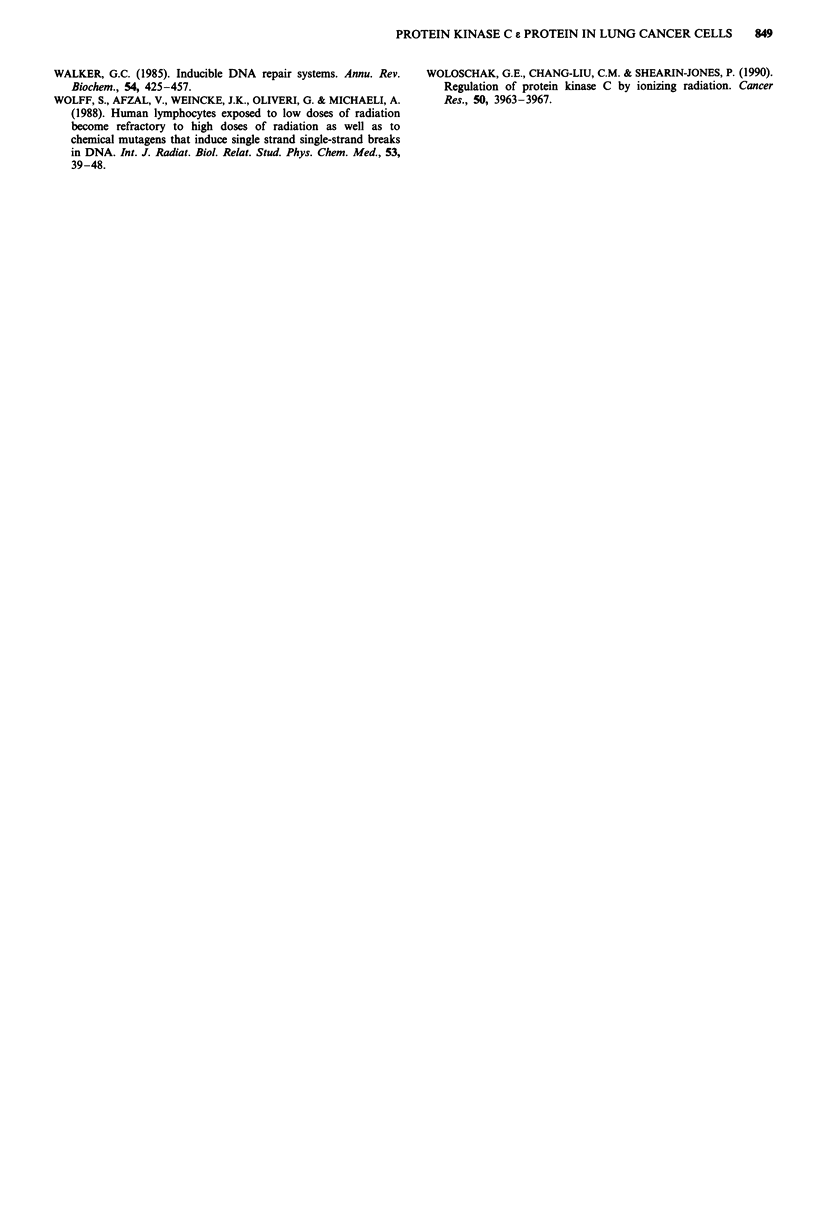# Differential expression of protein kinase C epsilon protein in lung cancer cell lines by ionising radiation.

**DOI:** 10.1038/bjc.1992.371

**Published:** 1992-11

**Authors:** C. Y. Kim, A. J. Giaccia, B. Strulovici, J. M. Brown

**Affiliations:** Stanford University Medical Center, Department of Radiation Oncology, California 94305.

## Abstract

**Images:**


*Br. J. Cancer* (1992), **66**, 844–849 © Macmillan Press Ltd., 1992

### Differential expression of protein kinase C ε protein in lung cancer cell lines by ionising radiation

C.Y. Kim[1] A.J. Giaccia[1], B. Strulovici[2] & J.M. Brown[1]

[1]*Stanford University Medical Center Department of Radiation Oncology, Stanford California 94305 and* [2]*Syntex Corporation, Palo Alto, California, USA.*

**Summary** The effect of ionising radiation on the regulation of gene and protein expression is complex. This study focuses on the translational regulational of the epsilon isoform of protein kinase C by ionising radiation. We found that protein kinase C ε is rapidly increased in the human lung adenocarcinoma cell line A549 following irradiation. Western blots showed increased accumulation of this protein at doses as low as 75 cGy after 15 min post irradiation. Maximal induction (11-fold over unirradiated cells) of PKC ε occurred at 150 cGy within 1 h after treatment by X-rays in A549 cells. The increased levels of PKC ε protein after X-rays does not require *de novo* protein or RNA synthesis, suggesting that this increase is post-translationally controlled. In contrast to A549 cells PKC ε levels in the large cell lung carcinoma cell line NCI H661 were not induced by radiation. In the small cell lung carcinoma cell line NCI N417, PKC ε was also not induced but a higher molecular weight PKC ε protein, suggestive of phosphorylation, appeared at 2 h after irradiation. The variation in induction or phosphorylation of PKC ε by ionising radiation in the cell lines tested in this study suggested that no clear correlation existed between intrinsic radiation sensitivity and PKC ε induction. To determine whether PKC ε does play a role in cell survival to irradiation, we used the protein kinase inhibitor staurosporin to decrease PKC activity and found that staurosporin sensitised cells to killing by ionising radiation. Pulsed field gel electrophoresis, however, indicated that DNA double-strand break repair was not decreased, suggesting that PKC ε is modifying the fidelity of rejoining and not the overall magnitude of repair. The regulation of PKC by ionising radiation will be discussed with respect to the biological consequences of gene induction by DNA damage agents.

In prokaryotes ionising radiation induces a series of genes which are involved in DNA repair processes (Walker, 1985). For eukaryotic cells the data are still ambiguous as to whether the genes or proteins induced by DNA damaging agents are involved in DNA repair. Evidence for the presence of inducible DNA repair genes in mammalian cells comes from the work of Wolff *et al.* (Wolff *et al.*, 1988). They showed that if lymphocytes are pretreated with low doses of a DNA damaging agent such as X-rays or bleomycin, they become more resistant to cytogenetic damage when challenged with a higher dose of the same agent. Further evidence that inducible DNA repair processes exist in mammalian cells is found in the work of Boothman *et al.*, which demonstrates by two-dimensional gel electrophoresis that a specific set of proteins are induced when cells are irradiated and held under conditions which prevent them from cycling before they can repair their potentially lethal DNA damage (PLDR-Potentially Lethal Damage Repair) (Boothman *et al.*, 1989). Interestingly, one protein designated XIP 269 (X-ray inducible protein with an apparent molecular weight of 260 kilodaltons) showed a good correlation between its induction and the extent of PLDR in a variety of different cell lines. However, this protein is still unidentified.

The molecular trigger for the induction of genes and proteins by ionising radiation has not been elucidated but most probably involves the activation of pre-existing proteins since the kinetics of their activation is rapid. Sherman *et al.* have recently reported the induction of the proto-oncogenes c-*jun*, *jun* B anc c-*jun* by ionising radiation, suggesting that the damage produced by ionising radiation activates transcriptional regulators (Sherman *et al.*, 1990). UV light and other DNA damaging agents also induce c-*fos* and c-*jun*, implicating DNA damage as a cellular sensor for gene activation (Hollander & Fornace, 1989). Regulation of transcriptional factors, in general, is not dependent on *de novo* protein or RNA synthesis, indicating that post-translational modification of pre-existing proteins, such as phosphorylation, is responsible for their DNA binding activation. One example of a transcriptional factor activated by ionising radiation is the AP-1 protein. AP-1 is composed of homo or heterodimers of c-*fos* and c-*jun* which bind to DNA to stimulate transcription of genes responsive to phorbol esters and growth factors (Angel *et al.*, 1987). The similarity of the response of increased transcription of c-*jun*, *jun* B and AP-1 after ionising radiation and after growth factors suggests that they might occur through a common activation mechanism such as PKC.

Recently there has been strong evidence for the involvement of signal transduction pathways, especially those involving PKC, as the link between radiation damage and the activation of transcription factors. At present, genes activated by ionising radiation can be broadly classified as PKC dependent or PKC independent. PKC dependent genes respond relatively rapidly within minutes post irradiation (Hallahan *et al.*, 1991*b*) and PKC independent genes require at least 5 h for maximal activation (Fornace *et al.*, 1988; Papathanasiou *et al.*, 1991). Hallahan *et al.* have reported that both tumour necrosis factor (TNF) gene expression (Hallahan *et al.*, 1991*b*) as well as EGR-1 and JUN gene expression (Hallahan *et al.*, 1991*a*) are mediated by protein kinase C following radiation. These data further implicate PKC as a regulator of gene expression by ionising radiation. However, it still remains to be determined whether PKC is the master regulator of early gene responses.

Thus far, eight distinct isoforms of PKC have been identified, exhibiting tissue specific expression and differences in their mode of activation and substrate specificity (Coussens *et al.*). At the DNA level these genes share some sequence homologies and are genetically distinct as they are located on different chromosomes (Nishizuka, 1988). This study focuses on the epsilon (ε) isoform of protein kinase C which is found in brain (Heidenreich *et al.*, 1990), thymocytes (Strulovici *et al.*, 1990), and small cell carcinomas of the lung (Baxter *et al.*, 1992). To determine what effect ionising radiation has on the expression of the PKC ε isoform, we analysed the kinetics of PKC induction in a variety of human lung tumour cell lines following x-irradiation. In addition, we found that inhibition of PKC by staurosporin sensitised cells to X-rays without affecting their ability to repair DNA double strand breaks.

Received 2 March 1992; and in revised form 11 June 1992

### Materials and methods

### *Cell lines and culture conditions*

Human lung adenocarcinoma A549 cells were obtained from the American Type Culture Collection (ATCC) and routinely cultured in alpha MEM containing 10% foetal calf serum. The small cell lung carcinoma cell lines used in this study were: classic small cell carcinoma cell lines: NCI H69, NCI H209, NCI H345, NCI H146 and variant cell lines: NCI H82, NCI-N417, NCI H526, NCI 841, NCI-H524. The large cell line used was a NCI H661. These cell lines were established by Adi Gazdar and generously provided by Dr John Minna (NCI, Bethedsa, Md) and cultured in RPMI 1640 medium with 10% heat inactivated serum. These cell lines have been previously described by Carmichael *et al.* with respect to their radiation sensitivity (Carmichael *et al.*, 1989) and biochemical and morphological properties (Carney *et al.*, 1985; Gazdar *et al.*, 1985). To reduce the effects of serum on PKC induction, cells were refed 16 h before the experiment with alpha MEM or RPMI containing 0.1% BSA before induction experiments. To down regulate PKC, $10^{-7}$ M TPA was added for 16 h before irradiation. Cells were irradiated at room temperature using either a Torex 150D X-ray cabinet at a dose rate of 0.45 Gy $min^{-1}$ or a $^{137}Cs$ source at a dose rate of 310 Gy $min^{-1}$. To determine whether induction required *de novo* protein or RNA synthesis, cells were preincubated for 1 h with either 1 μg $ml^{-1}$ or cyclohexamide or 10 μg $ml^{-1}$ of actinomycin D before irradiation.

### *Protein isolation and immunoblotting*

Cytosolic and particulate extracts of cells were prepared as previously described by Heidenreich *et al.* by lysing $5 \times 10^6$ cells on ice in buffer containing 10% SDS, 5% β-mercaptoethanol, 10% glycerol in 25 mM Tris-HCl, pH 6.8, and then sonicating the cells (Sonifier cell disruptor model W185 Heat systems-Ultrasonics, Inc.) at setting 7 for four three-second intervals on ice (Heidenreich *et al.*, 1990).

Two hundred μg of the protein extract was boiled for 5 min and subjected to one dimensional SDS/page as described by Laemmli (1970). After electrophoresis, immunoblotting of the separated proteins onto nitrocellulose paper was performed overnight at 250 mA by the method of Towbin *et al.* using a Biorad blotting apparatus (Towbin *et al.*, 1979). For immunological detection of the PKC ε protein, polyclonal antibodies previously described by Strulovici *et al.* (1991) were used at a dilution of 1:300 (Heidenreich *et al.*, 1990). These antibodies specifically recognise the PKC ε isoform. PKC ε was confirmed as the major reacting antigen by including a lane on the gel of rat brain extract which is highly enriched for this isoform. Nonspecific sites were first blocked by incubating the nitrocellulose paper with 5% instant nonfat milk (Carnation) and 0.5% Tween 20 for 1 h at 37°C. After blocking the non-specific sites, the nitrocellulose membrane was incubated for 4 h with a 1:200 dilution of antisera in 5% Carnation instant non-fat milk, 0.5% Tween 20 buffer, washed twice in the same solution without antibody, and then incubated with 0.1 μCi $ml^{-1}$ of $^{125}I$-protein A (Amersham) for 1 h. After three more washes of 10 min each, the nitrocellulose paper was dried at room temperature, and exposed to Kodak XAR-1 film with intensifying screens at − 70° for 3 to 7 days. Quantitation of PKC induction was determined using a LKB 2272-020 ultrascan excel enhanced scanning laser densitometer with gel scan XL-software.

### *Radiation response*

Survival curves to assay for cellular radiation sensitivity were performed on cells that were depleted of serum for 16 h as described above. Cells were untreated or pre-treated 1.5 h before irradiation with varying concentrations of staurosporin (50–500 nM). Some cells also received an additional 1.5 h of staurosporin treatment post irradiation. All cells were then washed twice and plated in six-well plates (Costar). Clonogenic survival was determined after incubation at 37°C for 10 days by fixing colonies with 10% formalin and staining with 1% crystal violet solution for counting. Colonies of 50 cells or more were counted. Survival curve was generated from three experiments with three replicates per point.

### *Pulsed-field gel electrophoresis*

The procedure of Giaccia *et al.* (1990) for asymmetrical-field inverted gel electrophoresis was used to determine the effect of staurosporin on the rejoining rate of DNA double-strand breaks in A549 cells by determining their rejoining capacity after 2500 cGy. Briefly, $5 \times 10^5$ A549 cells were initially plated onto 100 $mm^2$ plates with 12 ml alpha MEM containing 10% FCS and 0.24 μCi $ml^{-1}$ methyl $^{14}C$-thymidine (40 mCi $mmole^{-1}$, Amersham). After 2–3 days, the cells were refed with alpha MEM containing 0.1% BSA and 0.24 μCi methyl $^{14}C$-thymidine per ml for 16–20 h. The cells were then washed with alpha MEM containing 0.1% BSA and incubated for 1.5 h in the same media to remove unincorporated radioactive thymidine. The cells were then incubated for 1.5 h with 500 nM staurosporin in alpha MEM containing 0.1% BSA, irradiated with 2500 cGy and then inbeded in 1% agarose (Insert agarose-FMC, Rockland, ME) following incubation at 37°C for 0, 0.5, 1.0, and 3.0 h after irradiation. For each experiment unirradiated and untreated controls were also included. The agarose solution containing the cells was cast into 3 mm diameter glass tubes, cut into 5 mm cylinders, and then lysed for 24 h in 10 volumes of lysis buffer (0.5 M EDTA pH 7.9, 1% Sarkosyl, 1 mg proteinase K $ml^{-1}$) (Boehringer Mannheim, Indianapolis, IN) at 50°C. After this lysis period, the plugs were then dialysed twice against 25–50 vol of TE (10 mM Tris pH 7.4, 1 mM EDTA) and then incubated for 1 h in TE containing 0.1 mg of ribonuclease A per ml (Boehringer-Mannheim). These RNA free plugs were then loaded into 2 mm wells of an 0.8% agarose gel and asymmetrically electrophoresed (Denko *et al.*, 1989) for 16–24 h. The conditions for AFIGE were 900 s at + 1.25 V $cm^{-1}$ and 75 s at − 5.0 V $cm^{-1}$. Under these conditions, marker chromosomes from *Schizosaccharomyces pombe* and *Saccharomyces cerevisea* of 5.7 Mb or less will enter the gel, while larger DNA fragments will not be eluted into the lane from the well. For quantitation, the wells were separated from the lanes by a scalpel and each individually placed into a scintillation vial. Before melting on the agarose, 50 ml of 10N HCl was added to prevent the agarose from repolymerising. Scintillation fluid was then added to the samples, and they were counted on a Beckman LS 6000IC scintillation counter. The % of DNA double-strand breaks rejoined was determined by the formula:

$$1 - \left\{ \frac{\text{(\% DNA released after treatment at time t)} - \text{(\% DNA released after no treatment at time t)}}{\text{(\% DNA released after treatment at 0 h)} - \text{(\% DNA released after no treatment at 0 h)}} \right\} \times 100$$

For untreated controls, the amount of DNA released from the wells ranged from 1–3%.

### Results

### *Induction of PKC epsilon*

Previous experiments of Woloschak *et al.* (Woloschak *et al.*, 1990) demonstrated that ionising radiation increased PKC β mRNA levels in syrian hamster embryo fibroblasts. This 4–6 fold increase in PKC resulted after 75 cGy of X-rays, a relatively low dose in relation to individual doses used in cancer treatment. It seemed a strong possibility to us that a second level of regulation by ionising radiation could occur at the translational level. Therefore, we analysed the induction of PKC epsilon protein in lung carcinoma cell lines using a specific antibody against this isoform.

The time course of PKC induction following irradiation with a single dose of 75 cGY was determined by Western blotting and is shown in Figure 1. The effect of ionising

**Figure 1** Western blot of time course of PKC epsilon induction by 75 cGy of ionising radiation in three lung carcinomas (A549, NCI H661, NCI N417). The phorbol ester TPA was used as a control to downregulate PKC epsilon levels. The marker was derived from mouse brain extract which is highly enriched for PKC epsilon. NCI N417 insert b is a lighter exposure of insert a to illustrate covalent modification seen at 2 and 3 h.

radiation on the PKC ε isoform in the adenocarcinoma cell line A549 was quite rapid, with maximum induction by 15 min after irradiation. After 2 h, the levels of PKC ε returned to those of untreated controls. In contrast to A549 cells, the levels of PKC ε were unaffected by ionising radiation in NCI H661 cells derived from a large cell carcinoma. A third lung carcinoma cell line NCI N417 which originated from a small cell lung carcinoma also showed no change in PKC induction by ionising radiation. However, we noticed the appearance of a higher molecular weight protein that was recognized by our antibody at 2 h post irradiation (NCI N417 b). The appearance of this band suggests a post-translational modification of the 90 kDa PKC ε protein, most probably phosphorylation.

Since the induction of PKC ε in A549 cells occurred at relatively low doses of ionising radiation, we investigated whether its induction was dose dependent. As seen in Figure 2, PKC epsilon induction increased linearly for 1 h following a dose of 150 cGY. Interestingly, the kinetics of induction after 225 cGy were similar to those at 75 cGy. This suggests that a maximum protein induction exists in A549 cells at relatively low doses. Most importantly, PKC ε is induced strongly and rapidly in this cell line.

*The effect of transcription or translation inhibitors on PKC induction*

Due to the rapid induction of PKC ε by ionising radiation in A549 cells, we investigated whether this induction required *de novo* RNA or protein synthesis. Pretreatment of cells with 10 $\mu g\ ml^{-1}$ of actinomycin D (total RNA synthesis reduced to 7% of untreated as measured by $^3$H-uridine incorporation) or with 1 $\mu g\ ml^{-1}$ cycloheximide (total protein synthesis reduced to 18% of untreated as measured by $^3$H-leucine incorporation) for 1 h, followed by 75 cGy resulted in no change in PKC induction, but did result in the appearance of a second higher molecular weight band (Figure 3). Although we have not determined the relationship between these two bands, it is most probable that the higher molecular weight band represents a post-translational modification (phosphorylation) of the protein.

*Biological effects of inhibition of protein kinases on survival and DNA double-strand break rejoining*

To determine the relationship between PKC induction and survival, we inhibited PKC activity by preincubating the cells with the microbial alkaloid staurosporine and assessed the effect it had on cellular sensitivity to killing by ionising radiation. Preincubation of cells for 1.5 h with varying concentrations of staurosporin (50–500 nM) followed by irradiation and a subsequent 1.5 h incubation resulted in a dose dependent decrease in cellular survival (Figure 4). The staurosporin treatment itself resulted in 14% decrease in survival at 50 and 100 nM concentrations, and a 28% decrease in survival at a 500 nM concentration. Analysis of the survival curve showed that staurosporin had little effect on the initial part of the survival curve (0–400 cGy), but decreased the slope at higher doses (400–800 cGy) of the curve. This data suggests that inhibition of PKC interferes with the cellular response to rather larger doses of ionising radiation, but not to small ones. This result is puzzling in light of the fact that the induction of PKC ε occurred at doses of 75 cGy. However, it must be noted that staurosporin is a general protein kinase inhibitor (Badwey *et al.*, 1991), and the possibility remains that the sensitisation that we saw may not necessarily be due to PKC inhibition alone.

Since staurosporin was able to sensitise cells to killing by ionising radiation, we used AFIGE to determine whether this sensitisation was the result of inhibition of DNA double strand break rejoining. Figure 5 shows the kinetics of DNA

**Figure 2** Time course of PKC epsilon induction in A549 cells as a function of varying doses of ionising radiation (75, 150, 225 cGy). The time course of induction is similar for the three doses, with peak induction at 75 cGy occurring in 15 min, at 150 cGy in 1 h, and at 225 cGy in 30 min.

**Figure 3** Western blot of the effect of inhibitors of RNA synthesis (10 $\mu g\ ml^{-1}$ Actinomycin D) and protein synthesis (1 $\mu g\ ml^{-1}$ Cyclohexamide) on the time (in hours) of induction of PKC epsilon in A549 after treatment with 75 cGy. NT = untreated. PKC levels were unaffected, suggesting that induction does not require *de novo* RNA or protein synthesis.

**Figure 4** The effect of a protein kinase inhibitor, staurosporin, on survival of A549 cells after treatment with 200, 400, and 800 cGy. A549 cells were pretreated for 1.5 h before irradiation. The 0, 50, 100 and 500 nM treatments were also post treated for 1.5 h after irradiation. The 500 nM-Post received no staurosporin post treatment. At 200 and 400 cGy there were no significant differences in survival fraction. At 800 cGy, however, surviving fraction decreased with increasing staurosporin concentration, suggesting that inhibition of protein kinases sensitised the cells to radiation.

**Figure 5** Rejoining kinetics of DNA double-strand breaks in A549 cells after 2500 cGy treated or untreated with 250 μM staurosporin.

double-strand break rejoining in A549 cells. Both treated and untreated cells have similar rejoining kinetics with a half time of 30 min. Therefore, the sensitisation of A549 cells to killing by staurosporin seems to be independent of DNA double-strand break rejoining, *per se*. It is possible that the fidelity of rejoining is altered in the staurosporin treated cells. To determine if the fidelity of rejoining is grossly altered, chromosome aberration studies will be required.

Finally, to determine whether a correlation exists between radiosensitivity and PKC ε induction, we compared the surviving fraction after 200 cGy, to both steady state PKC ε levels and induction of PKC ε 1 h after radiation in 11 lung cell lines. The cell lines in this study failed to demonstrate a relationship between surviving fraction and either constitutive or inducible PKC ε levels (Table I). Other than A549, one other cell line, H82, a variant cell line, was found to exhibit significant induction. Of note this cell line has undetectable levels of constitutive PKC ε protein.

**Table I** Relationship between radiosensitivity and PKC ε induction

| *Small cell*[a] | *SF 2GY*[b] | *PKC ε*[c] | *Induction*[d] |
|---|---|---|---|
| *Classic* | | | |
| NCI H69 | 0.23 | + + | — |
| NCI H209 | 0.37 | + | — |
| NCI H345 | 0.21 | + + | N.D.[e] |
| NCI H146 | 0.066 | + + + | — |
| *Variant* | | | |
| NCI N417 | 0.56 | + + + | — |
| NCI H82 | 0.73 | − | 4-fold |
| NCI H526 | 0.72 | + | — |
| NCI 841 | 0.85 | + + | N.D. |
| NCI H524 | not done | + | — |
| *Large cell* | | | |
| NCI H661 | 0.93 | + | — |
| *Adenocarcinoma* | | | |
| A549 | 0.82 | + | 5-fold |

[a]Classification of tumour histology has been previously described in Carney *et al.*, 1985, *Cancer Res.*, **45**, 2913–2923 and Gazadar *et al.*, 1985, *Cancer Res*,. **45**, 2924–2929. [b]Radiation sensitivity of human lung cancer cell lines derived from Carmichael *et al.*, 1989, *Eur. J. Cancer Clin Oncol.*, **25**, 527–534. Surviving fraction following a 200 cGy dose. [c]The relative level of PKC ε protein cross reacting with antibody. The steady state levels of PKC epsilon were compared intraexperimentally with one another and grouped according to their relative levels (−, +, + +, + + +). [d]Induction of PKC ε protein at 1 h post radiation with 75 cGY. — indicates no induction. [e]N.D. = Not Determined.

### Discussion

In this paper we have described the transient accumulation of the PKC ε protein by ionising radiation in the A549 cell line. PKC ε induction occurred at low doses of ionising radiation within 15 min post irradiation suggesting that PKC induction might be useful as a biomarker/biodosimeter for ionizing radiation treatment in some tumour cell types, especially since the dose range for its induction is in the range of doses given in fractionated radiotherapy (100–200 cGy). Specifically, its usefulness as a biomarker could be applied to sections of tumour biopsies with polyclonal antibodies that recognise common epitopes for all PKC isotypes. Alternately, the polymerase chain reaction could be used to detect increased levels of PKC mRNA. Although PKC induction would be useful as a biomarker, the relationship between its induction and intrinsic cellular radiosensitivity is unclear.

Hirai *et al.* found that some lung cancer cell lines derived from squamous cell carcinomas, small cell carcinomas, or adenocarcinomas exhibit high levels of PKC activity compared to malignancies found at other sites (Hirai *et al.*, 1989). Lung carcinomas in general are responsive to radiation therapy, with the small cell lung cancer (SCLC) being more sensitive to radiation therapy than the other histological subtypes of lung cancer. Some cell lines derived from SCLC, however, exhibit a variant phenotype which may be more radioresistant than the classic phenotype (Carmichael *et al.*, 1989). Our data suggest that no clear correlation exists between PKC ε and surviving fraction. However, more work is needed to validate these results.

Interestingly, we found that the protein kinase inhibitor staurosporin is able to sensitise cells to killing by ionising radiation, seemingly independent of an effect on DNA double-strand break rejoining. To detect a difference in DNA double-strand break rejoining after treatment by staurosporin, we used the highly sensitive method of pulsed field gel electrophoresis. Since we saw no difference in DNA rejoining ability between staurosporin treated and untreated cells, we may assume that staurosporin sensitises cells to killing by ionising radiation by increasing DNA misrejoining, leading to higher levels of chromosome aberrations. This hypothesis will be tested by analysing chromosome aberrations induced by ionising radiation in treated *vs* untreated cells. However, it is possible that staurosporin is not affecting DNA/chromosome rejoining directly, but is altering cell cycle progression or $G_2$-phase delay. These possibilities are more likely as staurosporin is a general protein kinase inhibitor (Badwey et al., 1991) and many cell-cycle regulated events are mediated by protein phosphorylation and dephosphorylation (Gould & Nurse, 1989). In addition, PKC induction may not necessarily be a positive event, since it has an important role in tumour promotion.

The various PKC isotypes have different activation requirements. Several groups have reported that phosphorylation of protein kinase C results in a lower $K_a$ for $Ca^{2+}$ and a higher affinity for binding phorbol esters (Huang et al., 1986; Fry et al., 1985). Recently, Molina and Ashendel have demonstrated PKC phosphorylation in cells by treatment with TPA or DiC8 (sn – 1,2-dioctanoylglycerol), direct activators of PKC (Molina & Ashendel, 1991). These results imply that post-translational modification of PKC is important for its activation. We have also found that PKC ε is post-translationally modified by ionising radiation. This post translational modification was most clearly seen when cells were treated with actinomycin D or cyclohexamide before and after irradiation. Although we have not confirmed this higher molecular weight protein as the phosphorylated form of PKC ε, it has the same apparent molecular weight of the phosphorylated form of PKC ε as previously described by Pfeffer et al. (Pfeffer et al., 1991). Since this post-translational modification was seen when transcription and translation were inhibited, we propose that a protease exists which normally degrades or inhibits the function of this post-translational modification enzyme. Thus, the induction and post-translational modification of PKC act in concert to mediate cellular response to radiation damage. Further investigation is required to find the physiological significance of these responses.

This work was supported by U.S.P.H.S. Grant CA 15201 from the National Cancer Institute, D.H.H.S. to J.M.B., an institutional American Cancer Society grant and Grant CA 3353 to A.J.G., and the Syntex Research Group (B.S.). We would like to thank S. Daniel Issakani for characterising and purifying PKC ε. C.Y.K. is a James Ewing Foundation student fellow and Stanford Medical Scholar. We would like to thank Mrs Chiyoye Adachi for typing the manuscript, and Ms Mary Jean Clauss for critical reading of the manuscript.

### References

ANGEL, P., IMAGAWA, M., CHIU, R., STEIN, B., IMBRA, R.J., RAHMSDORF, H.J., JONAT, C., HERRLICH, P. & KARIN, M. (1987). Phorbol ester-inducible genes contain a common cis element recognized by a TPA-modulated trans-acting factor. *Cell*, **49**, 729–739.

BADWEY, J.A., ERICKSON, R.W. & CURNUTTE, J.T. (1991). Staurosporine inhibits the soluble and membrane-bound protein tyrosine kinases of human neutrophils. *Biochem. Biophys. Res. Commun.*, **178**, 423–429.

BAXTER, G., OTO, E., DANIEL-ISSAKANI, S. & STRULOVICI, B. (1992). Constitutive presence of a catalytic fragment of PKC e in a SCLC cell line. *JBC*, (in press).

BOOTHMAN, D.A., BOUVARD, I. & HUGHES, E.N. (1989). Identification and characterization of X-ray induced proteins in human cells. *Cancer Res.*, **49**, 2871–2878.

CARMICHAEL, J., DEGRAFF, W.G., GAMSON, J., RUSSO, D., GAZDAR, A.F., LEVITT, M.L., MINNA, J.D. & MITCHELL, J.B. (1989). Radiation sensitivity of human lung cancer cell lines. *Eur. J. Cancer Clin. Oncol.*, **25**, 527–534.

CARNEY, D.N., GAZDAR, A.F., BEPLER, G., GUCCION, J.G., MARANGOS, P.J., MOODY, T.W., ZWEIG, M.A. & MINNA, J.D. (1985). Establishment and identification of small cell lung cancer cell lines having classic and variant features. *Cancer Res.*, **45**, 2913–2923.

COUSSENS, L., PARKER, P.J., RHEE, L., YANG-FENG, T.L., CHEN, E., WATERFIELD, M.D., FRANCKE, U. & ULLRICH, A. (1986). Multiple, distinct forms of bovine and human protein kinase C suggest diversity in cellular signaling pathways. *Science*, **233**, 859–866.

DENKO, N., PETERS, B., GIACCIA, A. & STAMATO, T. (1989). An asymmetric field inversion gel electrophoresis system for the separation of large DNAs. *Anal. Biochem.*, **178**, 172–176.

FORNACE, A., ALAMO, I. & HOLLANDER, M.C. (1988). DNA damage-inducible transcripts in mammalian cells. *Proc. Natl Acad. Sci. USA*, **85**, 8800–8804.

FRY, M.J., GEBHARDT, A., PARKER, P. & FOULKES, J.G. (1985). Phosphatidylinositol turnover and transformation of cells by Abelson murine leukemia virus. *EMBO J.*, **4**, 3173–3178.

GAZDAR, A.F., CARNEY, D.N., NAU, M.N. & MINNA, J.D. (1985). Characterization of variant subclasses of cell lines derived from small cell lung cancer having distinctive biochemical, morphological, and growth properties. *Cancer Res.*, **45**, 2924–2929.

GIACCIA, A.J., DENKO, N., MACLAREN, R., MIRMAN, D., WALDREN, C., HARTI, I. & STAMATO, T.D. (1990). Human Chromosome 5 Complements the DNA Double-Strand Break-Repair Deficiency and Gamma-Ray Sensitivity of the XR-1 Hamster Variant. *Am. J. Hum. Genet.*, **47**, 459–469.

GOULD, K.L. & NURSE, P. (1989). Tyrosine phosphorylation of fission yeast cdc2+ protein kinase regulates entry into mitosis. *Nature*, **342**, 39–45.

HALLAHAN, D.E., SUKHATME, V.P., SHERMAN, M.L., VIRUDACHALAM, S., KUFE, D. & WEICHSELBAUM, R.R. (1991*a*). Protein kinase C mediates X-ray inducibility of nuclear signal transducers EGR1 and JUN. *Proc. Natl Acad. Sci. USA*, **88**, 2156–2160.

HALLAHAN, D.E., VIRUDACHALAM, S., SHERMAN, M.L., HUBERMAN, E., KUFE, D.W. & WEICHSELBAUM, R.R. (1991*b*). Tumor necrosis factor gene expression is mediated by protein kinase C following activation by ionizing radiation. *Cancer Res.*, **51**, 4565–4569.

HEIDENREICH, K.A., TOLEDO, S.P., BRUNTON, L.L., WATSON, M.J., DANIEL-ISSAKANI, S. & STRULOVICI, B. (1990). Insulin stimulates the activity of a novel protein kinase C, PKC-epsilon, in cultured fetal chick neurons. *J. Biol. Chem.*, **265**, 15076–15082.

HIRAI, M., GAMOU, S., KOBAYASHI, M. & SHIMIZU, N. (1989). Lung Cancer Cells often express high levels of protein kinase C activity. *Jpn. J. Cancer Res.*, **80**, 204–208.

HOLLANDER, C.M. & FORNACE, A.J. (1989). Induction of fos RNA by DNA-damaging agents. *Cancer Res.*, **49**, 1687–1693.

HUANG, K., CHAN, K.J., SINGH, T.J., NAKABAYASHI, H. & HUANG, F.L. (1986). Autophosphorylation of rat brain Ca2+-activated and phospholipid-dependent protein kinase. *J. Biol. Chem.*, **261**, 12134–12140.

LAEMMLI, U. (1970). Cleavage of structural proteins during assembly of the head of bacteriophage T4. *Nature*, **227**, 680–685.

MOLINA, C. & ASHENDEL, C. (1991). Tumor promoter 12-*O*-tetradecanoylglycerol increase the phosphorylation of protein kinase C in cells. *Can. Res.*, **51**, 4624–30.

NISHIZUKA, Y. (1988). The molecular heterogeneity of protein kinase C and its implications for cellular regulation. *Nature*, **334**, 661–665.

PAPATHANASIOU, M.A., KERR, N.C.H., ROBBINS, J.H., ALAMO, I., BARRETT, S.F., HICKSON, I.D. & FORNACE, A.J. (1991). Induction by ionizing radiation of a protein kinase C-unresponsive gene: gadd45. *Proc. Natl Acad. Sci. USA*,

PFEFFER, L.M., EISENKRAFT, B.L., REICH, N.C., IMPROTA, T., BAXTER, G., DANIEL-ISSAKANI, ?. & STRULOVICI, B. (1991). Transmembrane signaling by interferon a involves diacylglycerol production and activation of the e isoform of protein kinase C in Daudi cells. *Proc. Natl Acad. Sci, USA*, **88**, 7988–7992.

SHERMAN, M.L., DATTA, R., HALLAHAN, D.E., WEICHSELBAUM, R.R. & KUFE, D.W. (1990). Ionizing radiation regulates expression of the c-*jun* protooncogene. *Proc. Natl. Acad. Sci. USA*, **87**, 5663–5666.

STRULOVICI, B., DANIEL-ISSAKANI, S., BAXTER, G., KNOPF, J., SULTZMAN, L., CHERWINSKI, H., NESTOR, J., WEBB, D.R. & RANSOM, J. (1990). Distinct mechanisms of regulation of protein kinase C by hormones and phorbol esters. *J. Biol. Chem.*, **266**, 168–173.

STRULOVICI, B., DANIEL-ISSAKANI, S., BAXTER, G., KNOPF, J., SULTZMAN, L., CHERWINSKI, H., NESTOR, J.J., WEBB, D. & RANSOM, J. (1991). Distinct mechanisms of regulation of protein kinase C epsilon by hormones and phorbol esters. *JBC*, **266**, 168–73.

TOWBIN, H., STAEHELIN, T. & GORDON, J. (1979). Electrophorectic transfer of proteins from polyacrylamide gels to nitrocellulose sheets: procedure and some applications. *Proc. Natl Acad. Sci, USA*, **76**, 4350–4354.

WALKER, G.C. (1985). Inducible DNA repair systems. *Annu. Rev. Biochem.*, **54**, 425–457.

WOLFF, S., AFZAL, V., WEINCKE, J.K., OLIVERI, G. & MICHAELI, A. (1988). Human lymphocytes exposed to low doses of radiation become refractory to high doses of radiation as well as to chemical mutagens that induce single strand single-strand breaks in DNA. *Int. J. Radiat. Biol. Relat. Stud. Phys. Chem. Med.*, **53**, 39–48.

WOLOSCHAK, G.E., CHANG-LIU, C.M. & SHEARIN-JONES, P. (1990). Regulation of protein kinase C by ionizing radiation. *Cancer Res.*, **50**, 3963–3967.